# Face processing in police service: the relationship between laboratory-based assessment of face processing abilities and performance in a real-world identity matching task

**DOI:** 10.1186/s41235-021-00317-x

**Published:** 2021-08-05

**Authors:** Markus M. Thielgen, Stefan Schade, Carolin Bosé

**Affiliations:** Department I – University Education, Area of Study VIII – Social Sciences, Rhineland-Palatinate Police University, Post Box 1111, 55482 Hahn-Airport, Germany

**Keywords:** Individual differences in face processing abilities, Laboratory-based tasks, Work samples, CCTV, Police services, Predictive validity, Personnel selection

## Abstract

In the present study, we investigated whether police officers’ performance in searching for unfamiliar faces in a video-based real-world task is predicted by laboratory-based face processing tests that are typically used to assess individual differences in face processing abilities. Specifically, perceptual performance in the field was operationalized via the identification of target individuals in self-made close-circuit television (CCTV) video tapes. Police officers’ abilities in the laboratory were measured by the Cambridge Face Memory Test long form (CFMT+). We hypothesized that the CFMT+ predicts individual differences in the CCTV task performance. A total of *N* = 186 police officers of the Rhineland-Palatinate State Police participated in the study (i.e., *N* = 139 novice and advanced cadets with either 3 months, 15 months or 24 months of pre-service experience; *N* = 47 experienced police officers with three years of pre-service experience and at least two years of full-service experience, who participated in the assessment center of the special police forces, specifically the surveillance and technical unit). Results revealed that the CFMT+ explained variance in the CCTV task. In sample 1, CFMT+ scores predicted hits, but not false alarms. In contrast, in sample 2, CFMT+ scores were correlated with both hits and false alarms. From a theoretical perspective, we discuss factors that might explain CCTV task performance. From a practical perspective, we recommend that personnel selection processes investigating individual differences of police officers’ face processing abilities should comprise of two steps. At first, laboratory-based tests of face processing abilities should be applied. Subsequently, to validate laboratory-based individual differences in face processing abilities, we recommend that work samples such as CCTV tasks from the field should be added.

**Supplementary Information:**

The online version contains supplementary material available at 10.1186/s41235-021-00317-x.

## Introduction

Recently, major crimes such as terror attacks or crowd events like the Cologne New Years’ Eve riots in 2015 challenged police organizations worldwide (e.g., Eddy, 2016). Basically, in order to promote both crime prevention and law enforcement, security-critical verification tasks covering crowd surveillance, passport authentication or criminal investigation are essential for police operations. Therefore, both human competencies and artificial intelligence are increasingly deployed. Regarding artificial intelligence, information technology has to take into account a wide range of requirements to develop a software of human face processing yielding high levels of accuracy (Davis et al., 2010). However, a recent pilot study at Berlin Südkreuz train station revealed that the current face processing software lacks to perform sufficiently and remained beyond expectations. Specifically, the applied technique still failed to achieve satisfactory accuracy levels and societal acceptance (Dahlkamp et al., 2020; cf. Murray & Fussey, 2019).

Regarding human competencies, several police organizations in different countries have tried to identify police officers with superior face processing abilities (Davis, 2019; Frankl, 2019). For instance, the London Metropolitan Police pioneered the first specialized unit of so-called super-recognizers in police organizations (Robertson et al., 2016). In terms of the science-practitioner divide model (Anderson et al., 2001), the research area of “super-recognizers” is quickly emerging in a short period of time from “popularist” science toward pragmatic science with both high practical relevance and high scientific rigor. However, a gap between science and practice might still exist to date, concerning the empirical evidence and the practical deployment of individuals with superior face processing abilities. In practice, super-recognizers might be considered as a distinct group of individuals with extraordinary face processing abilities. In research however, this categorization regarding face processing abilities seems to be inappropriate. Therefore, Moreton et al. (2019) urged for a greater collaboration between researchers and the applied community. Quite recently, a series of high-quality papers in the British Journal of Psychology headed by Ramon et al. (2019a) addressed the debate with respect to super-recognizers, encouraging research “from the lab to the world and back again” (p. 461).

Summarizing the debate, it seems to be crucial to understand the relationship between individual differences measured by laboratory-based face processing tests and performance in real-world police tasks. Although research of face processing has been worked on for decades (e.g., Bruce, 1979, 1982; Carey & Diamond, 1977; Ellis, 1975; Ellis et al., 1979; Sporer, 1992), the investigation of superior face processing skills (performed by individuals tagged as “super-recognizers”) was initiated quite recently (Russell et al., 2009, 2012). Actually, a limited number of empirical studies provided little empirical insights with scarce theoretical and methodological progress (Ramon et al., 2019a). So far, it is accepted that individual differences in face processing abilities can be described as a quantitative continuum reaching from developmental prosopagnosia to super-recognizers (Bobak et al. 2016e; Russell et al., 2009; Tardif et al., 2018; Wang et al., 2012). Methodically, existing research had predominately identified individuals with superior face processing performance by distinct laboratory-based psychometric tests originally not designed for that purpose (Fysh, 2018; Fysh et al., 2020; Stacchi et al., 2020). In sum, it seems to be important to understand the relationship between ability and performance across the whole continuum both theoretically and methodically, instead of focusing on “super-recognizers” identified by laboratory-based tests solely.

In this context, police officers are not deployed in distinct laboratory tasks, but engaged in complex real-world tasks such as crowd surveillance, passport authentication and criminal investigation (Davis & Valentine, 2008; Ramon, 2019; Ramon et al., 2019a; White et al., 2014). Thus, the question whether individual differences in laboratory-based face processing ability tests transfer into complex real-world tasks is still unacknowledged (Ramon et al., 2019b; Stacchi et al., 2020; Towler et al., 2017). Indeed, a solid understanding of individual differences in face processing abilities and performance in real-world face matching tasks by police officers would be a prerequisite for an empirically based personnel selection process.

## Theoretical background

### The importance of face processing ability

Perception, processing and recognition of faces are core phenomena of mental activity (Leopold & Rhodes, 2010). Herein, faces can be seen as “One of the richest and most powerful tools in social communication […]. Specifically, humans may infer information from faces such as identity, gender, sex, age, race, ethnicity, sexual orientation, physical health, attractiveness, emotional state, personality traits, pain or physical pleasure, deception, and social status.” (Jack & Schyns, 2015, p. 621). From an evolutionary perspective, face processing seems to be a unique cognitive process that is genetically based and rapidly developed. Indeed, the ability to remember thousands of faces as “unique” seems to be evolutionary adaptive (Burke & Sulikowski, 2013; Maguinness & Newell, 2014).

One might assume that most people perform well on identifying faces (Young & Burton, 2017). Indeed, recognizing familiar people seems to be easy, even under adverse and restricted perceptual conditions, e.g., in the dark (Jenkins et al., 2011; Young & Burton, 2017). Here, the ability of processing familiar faces refers to the identification of well-known people by faces that had been seen frequently before in different situations, whereas different types of information derived from the face were integrated (Bruce & Young, 1986; Burton et al., 1999). However, the processing of familiar faces does not generalize well to unfamiliar faces that had been seen only once or a few times before (Johnston & Edmonds, 2009). Experimental research on face processing was initiated by Ellis (1975). Typically, subjects are asked to regard pictures of unfamiliar faces for a short period of time. Subsequently, they have to recognize pictures of the learned faces among a series of photographs presenting different target and distractor faces (Bruce, 1979, 1982; Ellis, 1975; Johnston & Edmonds, 2009; Longmore et al., 2008; Young & Burton, 2017). Results of experimental investigation confirm that performance in familiar face processing is usually easier than unfamiliar face processing. Finally, research shows that general and specific factors are involved, i.e., ability to perceive, to process, to discriminate and to recognize unfamiliar faces (Verhallen et al., 2017).

In law enforcement, research on eyewitness testimony suggests that processing of familiar faces is quite accurate, whereas errors in processing unfamiliar faces and identification are highly frequent (Wells & Olson, 2003; Wells et al., 2002). The latter effect is particularly important, because eyewitness misidentifications are a major factor in miscarriages of justice (Brewer & Wells, 2011; Howe et al., 2018; Rattner, 1988; Sauer & Brewer, 2015; cf. Freiwald et al., 2017, for a review). In modern police services abilities in processing unfamiliar faces are involved in a wide range of tasks, including crowd surveillance, passport authentication or criminal investigation. Thus, we will further focus on processing of unfamiliar faces.

### Testing individual differences of face processing in the laboratory

Although processing of unfamiliar faces is a critical factor for human social behavior, several studies suggested substantial individual differences on a continuum from inferior to superior performance (e.g., Davis et al., 2016; Duchaine & Nakayama, 2005; Duchaine et al., 2007; Freiwald et al., 2017). Research on face processing originally aimed to investigate inferior performance of face cognition, i.e., prosopagnosia. Individuals with developmental prosopagnosia lack to perform in face processing sufficiently. They may fail to learn new faces, to recognize old faces and to distinguish between similar and different faces. More recently, individual differences in face processing abilities received significant research interest, particularly with focus on extraordinary performance, i.e., super-recognizers (Robertson et al., 2016; Yovel et al., 2014; cf. Russell et al., 2009). Compared to average performance of face processing, superior performers should be excellent in learning new faces, recognizing old faces and discriminating between faces of low or high similarity (Bobak et al., 2016b). Consequently, the so-called super-recognizers reveal a larger inversion effect when sorting pictures of inversed faces according to their similarity as compared to upright faces than individuals with average or impaired face processing (Duchaine & Nakayama, 2005; Duchaine et al., 2007; Russell et al., 2009).

In the research context, individual differences in face processing abilities have been predominately assessed by several laboratory-based psychometric measures. Specifically, these tests incorporated different underlying tasks (e.g., unfamiliar identity matching) and were used across different sub-populations (i.e., prosopagnosia, individuals with average face processing performance and super-recognizers; Bobak et al., 2016c; see Ramon et al., 2019a, for a review). One of the most common tests of face processing ability in the field are the Cambridge Face Memory Test (CFMT; Duchaine & Nakayama, 2005) and the CFMT Long form (CFMT+; Russell et al., 2009). Whereas the CFMT is suitable to differentiate individuals with prosopagnosia from those with average performance, the CFMT+ comprises additional trials with high item difficulty to screen individuals with superior face processing abilities. Thus, the CFMT+ is suitable to assess the whole continuum of face processing abilities (cf. Ramon et al., 2019a). In this regard, recent studies have demonstrated high variability in face processing abilities using different laboratory-based tests. For instance, Fysh et al. (2020) applied six face processing tests showing that individual differences may be described by a normally distributed continuum. Likewise, Stacchi et al. (2020) applied two more challenging laboratory-based face processing tests, i.e., the Yearbook Test (YBT; Bruck et al., 1991) and the Facial Identity Card Sorting Test (FICST; Jenkins et al., 2011), confirming high interindividual variability, even with difficult task material. Noteworthy, the aforementioned tests had been developed in the laboratory for research purposes (Ramon et al., 2019a, b). However, empirical evidence of ecological validity is rare to date. Besides laboratory-based tests, initial research developing ecologically valid measurement approaches seems to be promising, such as the Spot the Face in a Crowd Test (Davis et al., 2018; Mileva & Burton, 2019) or the checkpoint search test (Kramer et al., 2020).

### Testing individual differences of face processing in the police context

In the applied context, individual differences in face processing abilities are of particular interest, especially for institutions in the security sector. Police organizations usually aim to predict and maximize performance in real-world tasks involving perpetrator identification (Ramon, 2019). Concerning the assessment of individual differences in face processing abilities, subjects are typically tested by laboratory-based instruments, mostly the CFMT+. However, evidence on the link between specific test scores in laboratory-based psychometric measures and performance levels in real-world tasks in the police context is relatively rare (e.g., Davis et al., 2018; Fysh, 2018; Fysh et al., 2020; Ramon, 2019; Stacchi et al., 2020). Moreover, several laboratory-based face processing tests exist that had not yet been linked to real-world tasks sufficiently at all (Bate et al., 2018; Dunn et al., 2020; Fysh, 2018; Fysh et al., 2020), excepting the CFMT+ (e.g.; Davis et al., 2018). Finally, the testing material of laboratory-based tests was based on pictures with high resolution showing only faces excluding hair and clothing, appearing some kind of artificial. Thus, it is questioned whether performance on such tests generalizes to performance in the field. However, it is of particular interest how individual differences predict performance in applied police tasks. In order to clarify the validity of face processing in the police context, laboratory-based tests of face processing need to predict performance in real-world tasks sufficiently, e.g., CCTV tasks. In this context, Davis et al. (2018) investigated police officers having superior unfamiliar face processing abilities in suspect identification on CCTV material by applying the Spotting the Face in a Crowd Test. Results revealed that both super-recognizers and police identifiers (who are experienced in suspect identification from CCTV) from the special unit of the London Metropolitan Police outperformed trained and untrained control subjects in the Spot the Face in a Crowd Test. In addition, they were less susceptible to change blindness errors. In sum, individual differences in laboratory-based test performance of face processing ability seem to explain performance in real-world CCTV footage. However, further research is needed.

### The present study

The digital age offers many opportunities in both crime prevention and law enforcement to enhance public security. CCTV may help to clarify crime by documenting evidence of criminal acts reaching from minor crimes such as shoplifting to major crimes such as terrorist attacks (Ratcliffe et al., 2009). However, the analysis of CCTV material often incorporates several obstacles. Concerning technical aspects, video tapes are often of poor quality. Apart from that, assessing CCTV material may be a time-consuming and labor-intensive task. For instance, during major events with large crowds such as political demonstrations, football games or music events a vast amount of tapes has to be analyzed. In addition, from an investigative perspective, it is important to identify potential offenders distinctly in order to clarify criminal acts justifiably.

Based on a meta-analysis of the CCTV review process, Hillstrom et al. (2008) specified factors that contribute to person identification. Here they pointed out that individual differences in assessors’ abilities of face processing are crucial. Whereas several attributes of peoples’ physical appearance such as clothes, beard or hairstyle are interchangeable, human faces are rather invariant. Since computer software for person identification yet lacks to perform sufficiently, police organizations are dependent on human abilities (Phillips et al., 2018). In police services, CCTV tasks particularly involve unfamiliar face processing. Usually police officers have to match pictures of faces with video material, in order to find target persons (i.e., unfamiliar identify matching). Since identifying unfamiliar faces is relatively difficult, individuals’ abilities in face processing are crucial for police services.

In this context, we were particularly interested to see whether laboratory-based face processing test performance predicts performance in a real-world task. As laboratory tasks, we chose the well-established CFMT+ (Russell et al., 2009). As a real-world task, we chose the task of person identification in CCTV. Since empirical evidence in this context is rare, we aimed to extend the initial research (Bate et al., 2018, 2019b; Davis et al., 2018; Mileva & Burton, 2019; Stacchi et al., 2020). For sure, the construction of a realistic CCTV task may have its own value, because it might be used as a work sample in personnel selection of individuals regarding their face processing abilities. Indeed, different diagnostic measures may be used in personnel selection. According to Schulers’ (2000) trimodal approach of personnel selection, the CFMT+ might be considered as a part of the testing approach, whereas the CCTV task constitutes a work sample following the simulation approach (Schuler, 2000). Here meta-analytic evidence has shown that both the testing approach and the simulation approach incrementally predict job performance (e.g., Schmidt & Hunter, 1998). Thus, the CCTV task might be incorporated to test batteries in order to assess face processing abilities in the police context more validly.

Following the approach of ecological validity, we predicted a positive relationship between CFMT+ scores and performance on the real-world CCTV task of person identification (main hypothesis).

## Method

### Sample

To test our hypotheses, we aimed to recruit police officers in duty. A priori, we estimated the appropriate sample size. Typically, effect sizes estimated in social and personality psychology surround *r* = .21 (Richard et al., 2003), i.e., ranging between small- and medium-sized effects (Cohen, 1988, 1992). However, applied studies concerning face processing are relatively rare to date. Moreover, effect sizes in existing research are relatively wide ranged (e.g., *r*^2^ = .03; Davis et al., 2018; *r*^2^ = .17, Balsdon et al., 2018). Thus, we expected to find a small effect size of *r*^2^ = .10 in the field. Subsequently, in order to detect this effect size, we needed to acquire *N* = 130 participants for multiple regression analysis with two predictors, assuming type 1 error probability of *α* = .05 and statistical power of 1 – *β* = .90.

In the present study, we acquired two samples of police officers. First, *N* = 142 police officer candidates from Rhineland-Palatinate Police University participated in the study. Due to missing data, *N* = 3 participants had to be excluded from the sample. Thus, *N* = 139 police officer candidates entered data analyses (*N* = 91 male, 65.5%; mean age *M* = 22.9, SD = 3.4, *range* 19–34 years). Since police officers of the Rhineland-Palatinate state police needed to achieve a Bachelors’ degree in “Police Services,” the sample was well educated (university degree: 6.5%; high school graduation [Abitur]: 73.4%; vocational diploma [Fachabitur]: 18.7%; other degree: 1.4%). Students had either 3 months, 15 months or 24 months of police experience. Students with either 15 or 24 months of experience passed police trainings and performed supervised pre-services in local police stations. According to the Dreyfus and Dreyfus (1980, 1991) step model of expertise, the first sample contained both novices and beginners. Students participated during their lecture period. As incentive, they received two hours of compensatory time-off for participation. Moreover, qualified feedback of students’ performance was offered.

The second sample comprised *N* = 47 full-service police officers joining the 4-day assessment center for the special police forces of the Rhineland-Palatinate State Police, i.e., the technical and surveillance unit (*N* = 40 male, 85.1%; mean age *M* = 29.5, SD = 4.7, *range* 24–42 years of age). All participants hold a Bachelors’ degree or equivalent in “Police Services” (three-year studies of policing, including police training and practical services in local police stations). They also performed at least two years of full service within a police department applying different employments of police work. According to the Dreyfus and Dreyfus (1980, 1991) step model of expertise, the second sample covered both competent and proficient police officers. The testing materials of the present study were embedded within the cognitive test battery during the assessment center. Specifically, the cognitive testing took place on the second day. Participants expected to be selected based on their performance, including face processing. Thus, we expected that all participants were highly motivated. However, the provided dataset was only used for research purposes without having any effect on personnel selection decisions. This procedure was discussed beforehand with the executives of the police special forces. Noteworthy, after passing the assessment center the subsequent special police forces education program had to be completed successfully to join the technical or surveillance unit.

Notably, the consideration of different sub-populations within the police context enables both generalization of our main hypothesis and replication of the results (cf. Simmons et al., 2011).

### Material

In the present study, we used both a laboratory-based test of face processing abilities and a real-world task of identity matching. Regarding the laboratory-based test, we adopted a well-established measure of face processing and face memory abilities (Tardif et al., 2018), i.e., the Cambridge Face Memory Test Long Form (CFMT+; Russell et al., 2009). This test had been used to assess individual differences in face processing abilities (e.g., Tardif et al., 2018; Davis, 2019; Bate et al., 2018, 2019b).

*Cambridge Face Memory Test Long Form* (CFMT+; Russell et al., 2009). The CFMT+ is a standardized laboratory-based test for investigating both face processing and face memory performance of identity matching. It comprises of a total of 102 trials of increasing item difficulty. Basically, in the CFMT+ participants are asked to memorize pictures of target faces. Subsequently, they have to recognize these targets among pictures of distractor faces. Pictures solely show peoples’ faces, while periphery attributes such as hair are shielded out (for details, see Russell et al., 2009).

Besides the CFMT+, we also applied the Cambridge Face Perception Test (CFPT; Duchaine et al., 2007), as a second standardized laboratory-based test to measure face processing abilities of identity matching, i.e., the ability to perceive differences between faces. The CFPT was administered as described by Duchaine et al. (2007). Due to shared stimulus material of laboratory-based tests, the CFMT+ was applied first, followed by the CFPT. The score of the CFPT indicated erroneous identity matches. Noteworthy, we ran the statistical analyses based on CFMT+ scores solely. The CFPT comprises of both upright faces and inverted faces, whereas the CFMT+ only consists of upright faces. Since upright faces usually occur in naturalistic scenes captured on CCTV material, we focus only on the CFMT+.

*Close-Circuit Television task* (CCTV task). In order to estimate face processing performance in an applied context, we implemented a so-called work sample. Basically, work samples are tasks representing a typical job demand of a specific profession (Schmidt & Hunter, 1998; Schuler, 2000). In the present study, we constructed a CCTV task, comprising an event sample of different naturalistic city scenes recorded on video, comparable to a crowd test (Bate et al., 2018, 2019b; Davis et al., 2018; Mileva & Burton, 2019; cf. Sackett et al., 2012). Conceptually, the underlying construct of the CCTV task comprised identity matching performance between pictures of target faces and videos showing targets. Methodologically, performance of identity matching of pictures and videos constitutes latent variables. Subsequently, the event sample of videos was a set of manifest items that could be used to estimate the latent variable. Specifically, the set of videos was considered as a scale with each video representing an item of the scale in order to apply principles of classical test theory, i.e., to estimate scale and item statistics (cf. Murphy & Davidshofer, 2005; cf. Sackett et al., 2012).

Overall, we sampled 15 videos. However, due to insufficient quality we had to delete two videos from the set. Since humans have a tendency to expect presence rather than absence of target identities in tasks of face processing, we only included videos containing targets (cf. Bate et al., 2018). Subsequently, we excluded two videos without a target individual. Thus, a total of 11 videos with targets were included in the CCTV task. The videos were recorded in public at frequented places in the city of Trier (2 × campus of the University of Trier; 9 × city center of Trier). In order to realize varied item difficulty, we manipulated the filmed setting of the videos across three different dimensions (cf. Table 1; Additional file 1: cf. supplementary Table 4): the number of target individuals (0, 1, or 2), the faces’ view of the target individuals (frontal or lateral) and the number of bystanders (< 10, 10–20, and > 20). The number of bystanders referred to the moment when the target individual appeared in the video. At this moment, the number of filmed bystanders with recognizable faces was counted. However, the number of filmed bystanders in the entire videos comprised an indefinite high number. Indeed, unknown numbers of bystander are a typical feature CCTV material from the field. Following the ecological approach (Bate et al., 2018, 2019b; Young & Burton, 2017), only targets were actors in the present study, while the surrounded visual scene was entirely naturalistic and not varied by the experimenters (Davis et al., 2018; Mileva & Burton, 2019).

**Table 1:** Hits, hit rates %, false alarms and item statistics of the videos of the CCTV task

Video	Number of target persons (targets)	View of target persons	Number of bystanders	Hits				False alarms (F.A.)		
				Hits	Hit rate %	Corrected item-scale	Correlation hits-CFMT+	False alarms	Corrected item-scale	Correlation F.A.-CFMT+
				M (SD)	M (SD)	r	r	M (SD)	r	r
C	1 (C)	Frontal	< 10	.70 (.46)	.70 (.46)	.29	.18*	.16 (.44)	.08	− .01
				.85 (.36)	.85 (.36)	.36	.18	.49 (.72)	.13	− .05
D	1 (B)	Frontal	10–20	.30 (.46)	.30 (.46)	.19	.12	.43 (.61)	.23	− .07
				.49 (.51)	.49 (.51)	.38	.15	.45 (.58)	.33	− .23
E	1 (F)	Frontal	> 20	.27 (.44)	.27 (.44)	.15	.06	.36 (.67)	.18	− .09
				.34 (.48)	.34 (.48)	.44	.31*	.47 (.88)	.34	− .33*
F	1 (D)	Lateral	< 10	.21 (.41)	.21 (.41)	.20	.03	.34 (.56)	.13	− .04
				.30 (.46)	.30 (.46)	.20	− .04	.51 (.62)	.38	− .27
G	2 (G, H)	Lateral	10–20	1.20 (.70)	.60 (.35)	.24	.16	.25 (.53)	.31	.01
				1.32 (.59)	.66 (.30)	.19	.35*	.36 (.49)	.35	− .15
H	2 (E, F)	Frontal	< 10	.65 (.56)	.33 (.28)	.14	.15	.27 (.54)	.34	− .04
				.87 (.45)	.44 (.22)	.25	.22	.53 (.75)	.47	− .39**
I	1 (H)	Frontal	10–20	.50 (.50)	.50 (.50)	.24	.09	.44 (.62)	.30	.04
				.74 (.44)	.74 (.44)	.34	− .08	.45 (.65)	.20	.05
J	2 (B, I)	Lateral	< 10	1.07 (.68)	.54 (.34)	.25	.16	.36 (.68)	.28	− .06
				1.21 (.59)	.61 (.29)	.34	.31*	.30 (.55)	.34	.08
K	2 (A, E)	Lateral	10–20	.79 (.68)	.40 (.34)	.25	.13	.46 (.65)	.28	− .05
				1.15 (.72)	.57 (.36)	.16	.09	.43 (.62)	.24	− .12
L	2 (D, I)	Frontal	> 20	1.23 (.66)	.62 (.33)	.33	.13	.23 (.46)	.23	− .00
				1.49 (.59)	.74 (.29)	.28	.14	.32 (.63)	.25	− .29*
M	2 (A, C)	Lateral	> 20	.32 (.48)	.16 (.24)	.18	.13	.52 (.65)	.13	.05
				.40 (.50)	.20 (.25)	.42	.19	.68 (.73)	.39	− .17

The first row displays results of the first sample (*N* = 139), and the second row of the second sample (*N* = 47). Significant correlations are marked with **p < .01; *p < .05, respectively. Moreover, we performed significance tests between groups (analyses of variance with repeated measures) to test for differences of the video manipulations. Lateral videos compared to frontal videos resulted in fewer hits (*F*_1,185_ = 26.89, *p* < .001, *η*^2^ = .13), and fewer false alarms (*F*_1,185_ = 5.70, *p* < .05, *η*^2^ = .03). However, videos with two target persons did not differ to videos with one target person with respect to hits (*F*_1,185_ = 2.92, *p* > .05, *η*^2^ = .02). Videos containing two targets revealed fewer false alarms than videos containing one target (*F*_1,185_ = 81.47, *p* < .001, *η*^2^ = .31). Videos containing more than 20 bystanders resulted in fewer hits compared to videos containing less than 20 and 10 to 20 bystanders (*F*_1,185_ = 22.04, *p* < .001, *η*^2^ = .11). Videos containing less than 10, 10 to 20, and more than 20 bystanders did not differ with respect to false alarms (*F*_1,185_ = 2.88, *p* > .05, *η*^2^ = .02)

**Fig. 1:**
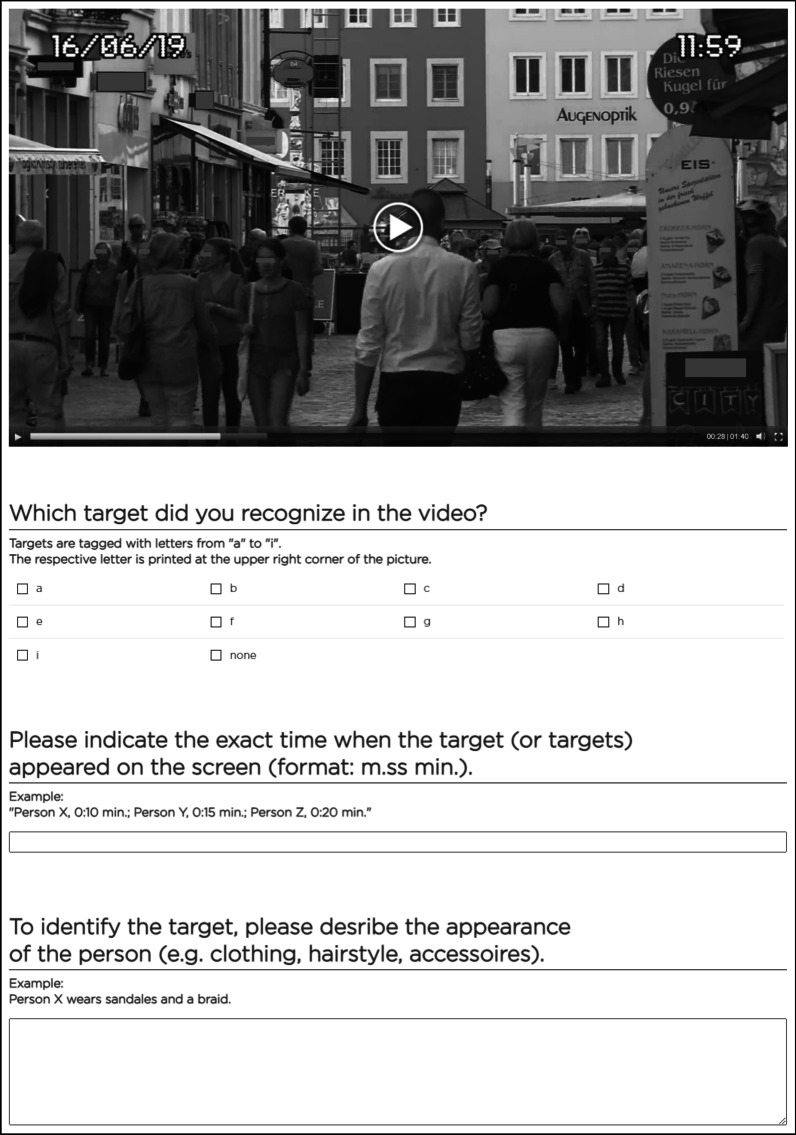
Example of CCTV task screen including the video. Note, in the study text was printed in German language

The videos were recorded by using a camcorder with full HD resolution. The camera was mounted on a tripod, such that the height of the objective lens was adjusted nearly to the eye line of an adult person. The height of the objective lens was kept constant across all filmed scenes. Subsequently, the videos were edited with video cutting software equalizing the play time constantly to 01:40 min per video. In addition, the filter mode “security” was applied in order to make videos appear like original CCTV files. Thus, the videos are depicted in black-and-white with time and date stamps placed in the upper corner of the video film (see Fig. 1).

**Fig. 2:**
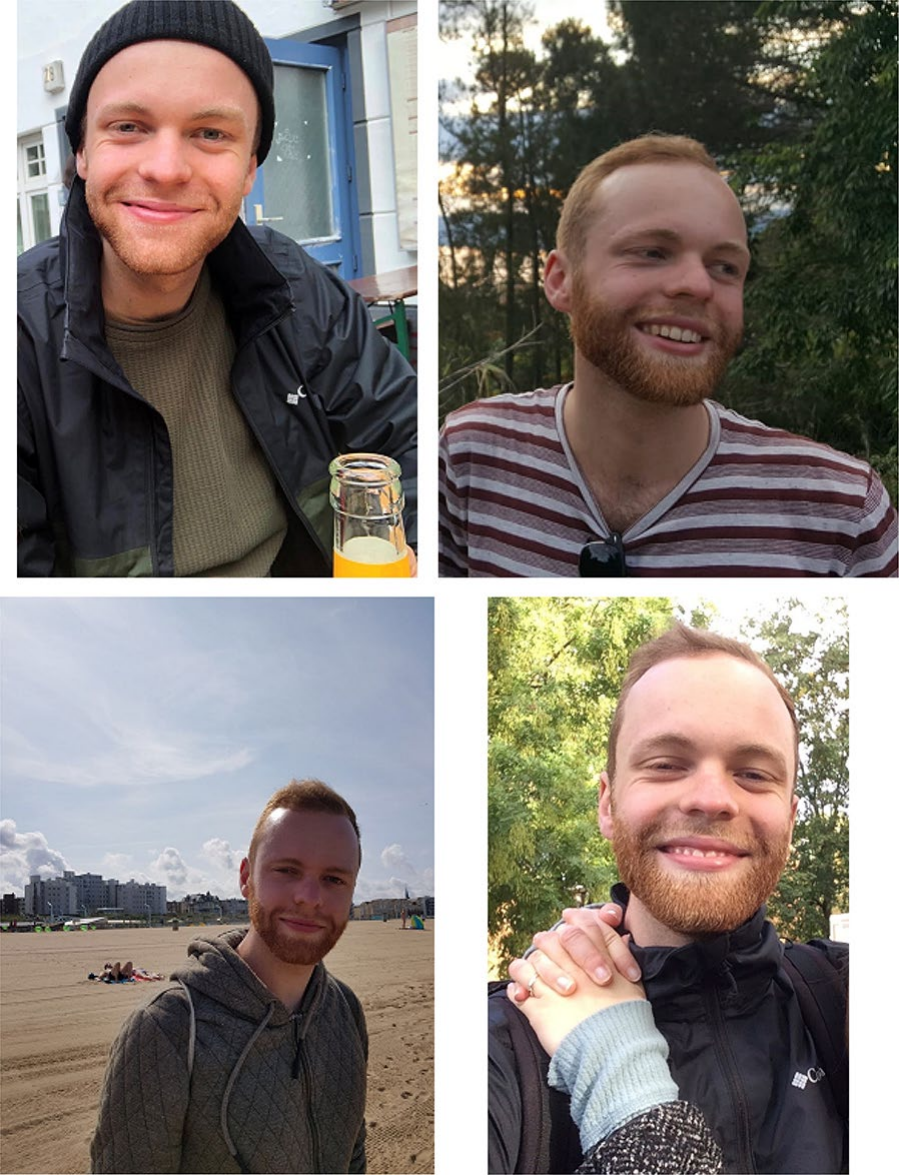
Example of individual photographs of a target person

As target persons, we recruited nine actors, coded with letters “a” to “i” (5 males, 20–27 years of age). Each actor appeared in two videos (except actor “G,” whose video had to be deleted due to insufficient quality of the video) for two reasons. Firstly, in criminal investigations several video files may be typically recorded, i.e., from different perspectives at different points in time. Subsequently, the same target might appear in several videos. Secondly, since a target occurs in two videos, we had the chance to compare the person statistics across the videos more reliably (cf. Additional file 1: Supplementary Table 4). Since the actors appeared in two different videos, they changed their physical appearance, e.g., by changing their clothes. Noteworthy, target individuals’ head and face were visible all the time when present in the video. Actors signed an informed consent that the video material could be used for the purpose of this study. To partially replicate Davis et al. (2018), we asked the actors to provide four individual photographs of themselves including both pictures of the actor’s face and the person at large (see Fig. 2). Here, actors were told that their photographs are best suitable if they would help police operations searching for missing people. All pictures were tagged with the id-codes of the target individuals from “a” to “i” and printed in color on DIN A4 paper format (210 × 297 mm).

At the beginning of the CCTV task, subjects were informed via instructions on the screen how to find targets in the videos. At first, participants were given a preparation phase. Subjects received the portfolio containing the printed pictures of all target persons. The preparation phase had two purposes. First, subjects were asked to get familiar with the target individuals. Second, they were asked to indicate whether they already know a target. One subject of the first sample admitted to know a target. The response of this subject to the respective video was codes as a “missing value.” In contrast, no subject of the second sample reported to know any target. In the first sample, subjects were allowed to review the pictures of the faces up to 3 min. In the second sample, subjects had no time limit for picture review. Here, they typically spent up to 15 min on the material. After the preparation phase, subjects started with the CCTV task. Notably, under the special circumstances of an assessment center, the aim was to realize rather a power test than a speed test. Moreover, in practical police service there is no time limit for reviewing pictures of potential suspects.

In the CCTV task, videos were presented via a web-based application (Unipark Enterprise Survey). The order of the videos was randomized per subjects (cf. Table 1). Each video was presented on a single screen (cf. Fig. 1). Here, subject watched videos with a media player. They were allowed to use all features of the media player, i.e., pausing or winding forward. Moreover, they could take notes on a notepad. While analyzing the videos, subjects were permitted to have a look in the portfolio with pictures of target faces at any time. In order to evaluate the CCTV material, subjects had to answer three questions. Firstly, subjects were asked to indicate the target person they recognized in the video by the respective letter “a” to “i" or “none.” If they recognized a target, they had to indicate both, the exact time when the target person appeared in the video, and the physical appearance of the target person. By this information, we verified the correct recognition of the target person. No time limit for the CCTV task was given. Notably, in the first sample the total time of the testing session was two hours, which was sufficient to complete both the laboratory-based tests and the CCTV task. In the second sample, the duration of both the laboratory-based tests and the real-world task lasted up to 2 h.

### Procedure

The study occurred in the first half of September 2019 (first sample) and in mid-January 2020 (second sample). Subjects were tested in a group setting in a computer cabinet for up to 20 individuals. At first, laboratory-based tests were administered. In this part, we administered the CFMT+ (Russell et al., 2009), followed by the Cambridge Face Perception Test (Duchaine et al., 2007). Due to the scope of the present paper, these data are not reported. Before testing, subjects were informed via screened instructions about the purpose of the study, i.e., to measure their face processing abilities and performance. Next, they answered the five questions regarding their subjectively rated face processing ability. Due to the scope of the paper, these data are not reported. The CFMT+ was administered as described by Russell et al. (2009). Finally, the CCTV task was administered.

### Scoring and statistical analysis

At first, we computed the scores of both the laboratory-based test and the real-world task. For the CFMT+, we computed one total score. For each target person that is correctly recognized subjects could receive one point. In total, subjects could receive between 0 and 102 points (for details, see Russell et al., 2009; Tardif et al., 2018).

Furthermore, we defined the scores of the CCTV task. Basically, the performance outcomes of the CCTV task were derived from signal detection theory (Green & Swets, 1966; Stanislaw & Todorov, 1999; Tanner & Swets, 1954) that are frequently used in research on face processing (e.g., Davis et al., 2018). First, a *hit* constituted a correct target identification, i.e., the target individual was present in the video and it was correctly recognized. The maximum number of hits that could be achieved was 17 with either 1 or 2 targets appearing in each video (see Table 1). Subsequently, the *hit rate* was constituted by the absolute number of hits divided by the maximum number of hits (cf. Table 1). This definition is in line with principles of signal detection theory (Green & Swets, 1966; Stanislaw & Todorov, 1999; Tanner & Swets, 1954; cf. Davis et al., 2018). Notably, since individuals had to indicate the exact time when the target person appeared in the video, the physical appearance and the walking direction of the target person, we could verify that a reported hit is truly a hit. Second, a *false alarm* constituted a misidentification, i.e., a subject erroneously identifies any bystander as a target. Remarkably, as the maximum number of bystanders in the videos was unknown, it was not possible to calculate a false alarm rate. In sum, the concepts hits, hit rates and false alarms in the CCTV tasks are comparable to signal detection theory. However, since the total number of bystanders was unknown, a false alarm rate analogous to signal detection theory could not be calculated. Thus, calculation of sensitivity (d’, hit rate—false alarm rate) and response bias (hit rate/false alarm rate) were not possible (Davis et al., 2018).

Concerning the statistical analysis, descriptive statistics and bivariate correlations of all variables of interest were calculated. In order to test our main hypothesis, we applied regression analyses by regressing CCTV task performance scores on CFMT+ test scores. Noteworthy, since the two samples were tested in different contexts (sample 1: study context vs. sample 2: personnel selection context), and two samples comprised different groups of police officers (sample 1: police officer candidates vs. sample 2: experienced police officers), we ran two separate analyses.

## Results

**Fig. 3:**
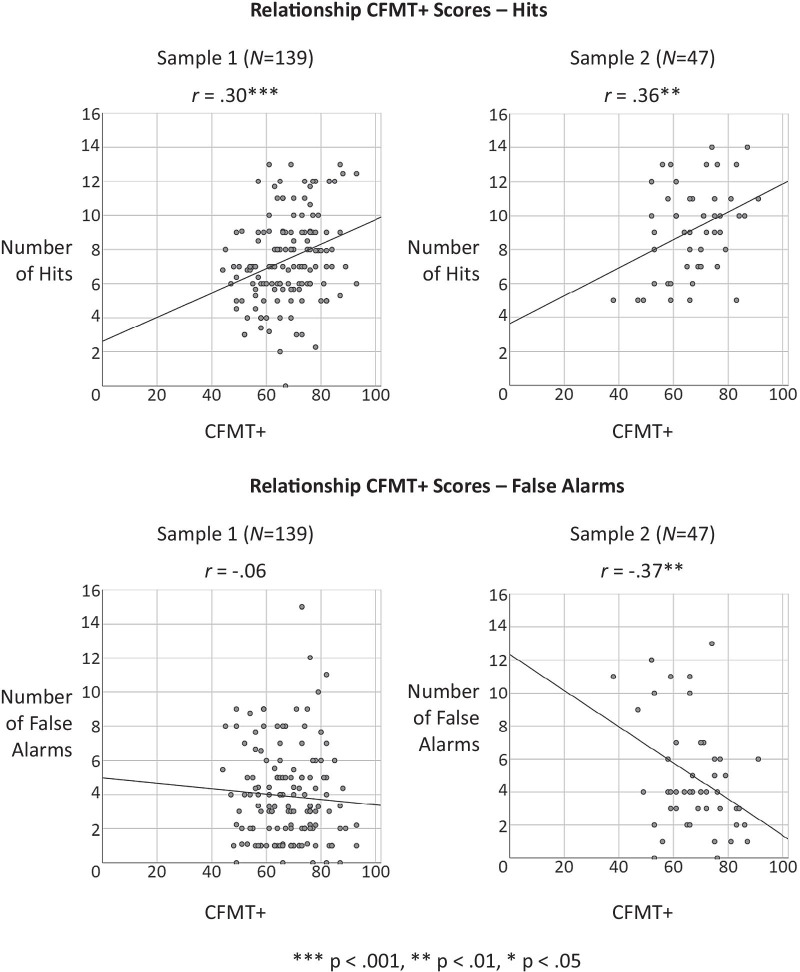
Scatterplots

Descriptive statistics, corrected item-scale correlations and bivariate correlations between the video-related performance scores, i.e., hits and false alarms, and laboratory-based test scores (CFMT+ scores), are presented in Table 1. Descriptive statistics of the variables of interest are displayed in Table 2. Bivariate correlations of all variables are reported in Table 3. Scatterplots are shown in Fig. 3.

**Table 2:** Descriptive statistics

Variable	Min	Max	*M*	SD	Skewness		Kurtosis	
						SD		SD
Age	19.00	34.00	22.86	3.39	1.36	0.21	1.35	0.41
	24.00	42.00	29.53	4.69	1.55	0.35	2.13	0.68
CFMT+	44.00	93.00	67.65	10.98	0.02	0.21	− 0.63	0.41
	38.00	91.00	67.21	11.77	− 0.14	0.35	− 0.38	0.68
*Target videos*								
Hits	0.00	13.00	7.43	2.61	0.16	0.21	− 0.17	0.41
	5.00	14.00	9.17	2.68	− 0.02	0.35	− 0.96	0.68
Hit rate %	0.00	0.76	0.43	0.15	0.16	0.026	− 0.17	0.41
	0.29	0.82	0.53	0.16	− 0.02	0.35	− 0.96	0.68
False alarms	0,00	15.00	3.91	2.78	1.02	0.21	1.20	0.41
	0.00	13.00	4.98	3.45	0.89	0.35	− 0.20	0.68

The first row displays results of the first sample (*N* = 139), and the second row of the second sample (*N* = 47)

**Table 3:** Correlations

	Gender	Education	Age	CFMT+	Hits	False alarms
Gender	–	.05	.18*	.12	.02	− .16†
	–	–	.11	− .01	.27†	− .06
Education		–	− .01	− .08	.06	.12
		–	–	–	–	–
Age			–	.15†	.18*	− .04
			–	.05	− .07	.18
CFMT+				–	.30***	− .06
				–	.36*	− .37**
Hits					(.54)	− .17*
					(.65)	− .08
False alarms						(.54)
						(.65)

The first row displays results of the first sample (*N* = 139), and the second row of the second sample (*N* = 47). Reliabilities (Cronbach’s alphas; Cronbach, 1951) are displayed in the diagonal in parentheses. †*p* < .10 two-tailed. *p < .05 two-tailed. **p < .01 two-tailed. ***p < .001 two-tailed. (-) No correlation could be calculated, because all subjects have the same education in Sample 2, because all subjects have a Bachelor’s degree

Empirical studies of face processing revealed gender and age to be important covariates. For instance, individuals aged around 30 years generally outperform younger and older ones (e.g., Germine et al., 2011). Thus, we ran all tests of hypotheses including gender and age as control variables. However, the result pattern remained the same. Subsequently, we report all analyses without control variables.

The main hypothesis of the present study stated that performance on the CFMT+ positively predicted performance in the real-world CCTV task of person identification. Regarding the first sample (Fig. 3), the CFMT+ score positively predicted hits of targets (*r* = .30, *p* < .001, one tailed, 95% CI [.14, .46]), but not false alarms (*r* = − .06, *p* > .05, one-tailed, 95% CI − .23 .11]). The CFMT+ explains 9.0% of variance in hits and 0.4% of variance in false alarms. Regarding the second sample (Fig. 3), the CFMT+ score positively predicted hits of targets (*r* = .36, *p* < .01, one-tailed, 95% CI [.08, .64]), and it was negatively related to false alarms (*r* = − .37, *p* < .01, one-tailed, 95% CI [− .65, − .10]). The CFMT+ explains 13.2% of variance in hits and 14.0% of variance in false alarms. In sum, the hypothesis was supported in general. Individuals with higher CFMT+ scores showed superior performance in the CCTV task (cf. Fig. 3).^1^

The figures show the relationship between laboratory-based test scores, i.e., CFMT+ scores, and performance in the CCTV task, i.e., hits and false alarms.

## Discussion

In order to enhance public security, CCTV footage is used both in crime prevention and in law enforcement (Ratcliffe et al., 2009; Robertson et al., 2016). For instance, after the recent riots of Stuttgart in the night of June 20/21 2020 individuals identified as “super-recognizers” searched for suspects using CCTV material. Video files were taken directly from surveillance cameras in the city, self-made smartphone videos of witnesses or police footage of the riot night to help criminal investigation (Schattauer, 2020). Indeed, police organizations are highly interested to deploy police officers with superior face processing abilities (Robertson et al., 2016). However, the relationship of laboratory-based test results and performance in applied tasks has rarely been investigated. Thus, further evidence is needed on how performance on standardized psychometric measures of face processing abilities is related to applied identity matching tasks performed by police in their daily work. Such evidence would be desirable in order to establish profound personnel selection processes involving face processing abilities (e.g., Ramon et al., 2019a, b).

Conceptually, CCTV tasks particularly involve face processing abilities of unfamiliar faces, i.e., identity matching. Here, operating police officers have to match pictures of faces with corresponding video material in order to detect and identify target persons. In this context, it seems to be highly desirable for police organizations to investigate individual differences in humans’ face processing abilities in order to promote task performance. In the present study, we investigated the relationship between face processing ability test scores and identified matching performance measures in a typical police task. Hereby, it is questioned whether laboratory-based ability tests predict the performance in the real-world task. As laboratory task, we chose a well-established psychometric test of face processing, i.e., the CFMT+ (Russell et al., 2009). As a real-world task, we adopted the task of person identification as identity matching via police-like CCTV material. Since empirical evidence regarding the ecology of laboratory-based tests in the police context is relatively rare so far, the present study aimed to extend the initial research (e.g., Davis et al., 2018; Mileva & Burton, 2019; Stacchi et al., 2020). Based on our theoretical reasoning, we assumed that performance on face processing in the CFMT+ positively predicted performance in the real-world CCTV task of person identification. As predicted, we found a positive correlation between the CFMT+ scores and CCTV task performance measures of police officers. In sum, the present study revealed empirical evidence supporting the valid prediction of performance in ecological meaningful identity matching by laboratory-based test performance. Subsequently, our results are in line with Davis et al. (2018), who revealed initial evidence, that individual differences in face processing abilities of police officers are particularly associated with high performance in an applied Spot a Face in the Crowd Test.

Noteworthy, our hypothesis was supported in general, i.e., CFMT+ scores positively predicted CCTV performance. However, comparing both samples we found different result patterns. In sample 1, CFMT+ scores predicted hits, but not false alarms. In contrast, CFMT+ scores in sample 2 were correlated with both hits and false alarms. Several differences between the two samples might account for the different result patterns. Firstly, sample 1 comprises of novice and advanced police officer cadets, whereas sample 2 contains experienced police officers (cf. Dreyfus & Dreyfus, 1980, 1991). Subsequently, the two samples differ in both education and experience. Indeed, criminalistic thinking and decision making is an essential part of police training and studies in the German police (Hansjakob et al., 2020; Niegisch & Thielgen, 2018). For instance, police officers learn to incorporate both verifying information and falsifying information in order to derive criminalistic decisions. Moreover, experienced police officers might be more aware of the risk of making incorrect identifications, e.g., from CCTV. Secondly, individuals of sample 2 applied for the surveillance and technical unit. In this context, a core job demand of undercover police officers is to make correct person identifications of suspects. Here, police officers have to weigh up risks of false positives and false negatives. Making a false positive decision may have consequences such as arresting a bystander, which subsequently may impact the success of the police operation (Meissner et al., 2015; Vrij & Granhag, 2014). Thus, it is possible that individuals with superior performance in the experienced group may have been more cautious in their decisions, producing fewer false alarms when uncertain. Thirdly, since police officers of the second sample participated in an assessment center for special police forces, they might had been aware that both hits and false alarms are criteria to value their performance, which could have impact on the personnel selection decision as well.

Finally, the CCTV task used may diagnostically help to assess individual differences in face processing abilities. Both the construction of CCTV tasks and the usage of original CCTV material might be implemented as so-called work samples in the personnel selection process of police officers working on applied face identity matching. The Spot the Face in a Crowd Test (Davis et al., 2018; Mileva & Burton, 2019) and our CCTV task might exemplify work samples. According to Schuler (2000) the application of standardized psychometric test diagnostics, such as the CFMT+, incorporates the testing approach of personnel assessment, whereas CCTV tasks, seen as work samples, constitute the simulation approach. Meta-analytic evidence revealed that both the testing approach and the simulation approach incrementally predict job performance (e.g., Schmidt & Hunter, 1998). Thus, the CCTV task might be incorporated to a test battery in order to select individuals high performing on face processing in the police context more validly. Indeed, CCTV tasks are usually complex and therefore might require a wide range of cognitive abilities, presumably not captured by the CFMT+.

### Limitations and implications for future research

The present study incorporated several limitations we discuss in the following section covering age effects, material, learning, motivation, social and contextual factors. Regarding human abilities of face processing, possible moderating effects may be considered. For instance, age-related effects on individual differences of face processing need to be investigated among police officers. Specifically, research suggests that face processing abilities seem to peak in the mid-30s. In this regard, both cross-sectional and longitudinal studies need to reveal age effects on face processing abilities across the occupational life span (e.g., Germine et al., 2011).

The nature of the material used has to be taken into account with respect to the quality of the material due to technical aspects (e.g., resolution, camera position), acting aspects (e.g., pose, expression) and the degree of naturalism of the visual scene (Young & Burton, 2017). For instance, if CCTV is from above head height, the likelihood of identification might be reduced. Thus, future research should systematically explore possible moderator effects of material on the relationship between test scores in laboratory-based tests and performance in real-world tasks (cf. Mileva & Burton, 2019). Likewise, Jenkins et al. (2011) suggest to study the naturally occurring images of faces, i.e., “ambient images” of faces (Bruce, 1994; Bruce & Young, 2012; Burton, 2013; Sutherland et al., 2013; Vernon et al., 2014).

Besides humans’ abilities of face processing, individual differences of identity matching performance may also rely on learning and motivation. Regarding learning factors, in research there is an ongoing debate whether operational factors such as job training or job experience are associated with higher performance in identity matching (Davis et al., 2018; Tree et al., 2017; Wilkinson & Evans, 2009; Wirth & Carbon, 2017). In this context, the cognitive involvement may predominately refer to the extent of how deeply participants process the graphical material of the target persons. If so, the nature of learning conditions might be relevant for improving performance. Consequently, future research has to take systematically into account the cognitive involvement with the learning material and different learning methods applied (Phillips et al., 2018). Considering learning aspects form a practitioner perspective, it is of particular interest whether training effects can be obtained in applied police tasks. Noteworthy, the categorical distinction between unfamiliar and familiar face processing might not be that distinctive. The underlying process of familiarization seems to be rarely understood to date (Devue et al., 2019; Ramon & Gobbini, 2018). Thus, factors that might facilitate or hinder the transition from unfamiliar to familiar faces need further investigation. In this context, a recent study evaluated existing training programs on face processing. Results revealed that trainings are yet limited to facial-image-comparison. Contrarily, facial-video-comparisons seem to be neglected so far. Hence, future research needs to specify the underlying processing strategies used in CCTV tasks (Towler et al., 2019). Regarding motivational factors, participants’ individual engagement in CCTV footage to identify target individuals correctly may play an important role in identity matching performance, irrespectively, of humans’ abilities of face processing.

Regarding social factors, it is questioned whether and how face processing performance is typically biased. For instance, individuals are usually better in processing faces from their own ethnicity as compared to other ethnicities. This other-ethnicity bias seems to be crucial for the police because investigative police officers typically aim to search for suspected target persons possessing different ethnicities and nationalities. Indeed, initial evidence substantially revealed the other-ethnicity bias among individuals with superior face processing abilities. However, they still outperform normal perceivers (Bate et al., 2019a). Specifically, evidence has shown that both identified super-recognizers and high-performing recognizers (not reaching test thresholds to be marked as “super”) achieve superior performance both in own- and other-ethnicity-tests of face processing (Robertson et al., 2019b). Likewise, a recent study also suggests other-age effects, i.e., individuals tend to be better at recognizing faces of their own age. Future research needs to address on how individual differences in face processing predict performance in real-world tasks with targets of different ages, i.e., children, adults and elderly people (Bate et al., 2020).

Contextual factors might be also taken into consideration. Both the Spot the Face in a Crowd Test (Davis et al., 2018; Mileva & Burton, 2019) and our CCTV task are identity matching tasks. These tasks seem to match classical visual search tasks. Visual search tasks are perceptual tasks requiring selective attention. Usually the environment is visually scanned for a specific target among several distractors (Treisman & Gelade, 1980; cf. Mackworth, 1948; Warm & Dember, 1998). Specifically, according to the guided search model proposed by Wolfe (1994) target features, e.g., features from faces, are actively used to guide selective attention throughout the visual environment (Wolfe, 1994, 2006). Notably these tasks are determined by several factors, i.e., target rarity (Wolfe et al., 2005), target numbers (Tickner & Poulton, 1975) and distractor frequency (Singh et al., 2007; Wickens et al., 2000). Both the Spot the Face in a Crowd Test used by Davis et al. (2018) and our CCTV task simultaneously present several targets and several distractors (i.e., bystanders) and other elements of the visual scene (e.g., houses, cf. Table 1). However, observation tasks in police practice resemble visual search tasks with low target frequency. A more recent study revealed that visual search efficiency seems to depend on whether visual search is conducted for either one or two unfamiliar faces (Mestry et al., 2017). Moreover, the learning material of target persons, e.g., within-face variability, may also affect visual search efficiency (Dunn et al., 2018). In sum, future research should take up the role of visual search mechanisms in searching for faces and individuals in real-world tasks.

Finally, recent literature on face processing used a value of 95 out of 102 on the CFMT+ for classification of super-recognition (< 2% of the population) (Bobak et al., 2016d; Noyes et al., 2021). However, none of our participants achieved scores beyond this threshold. Although this is an arbitrary standard, police officers achieving higher score level as observed in our study might also show highest performance scores in our real-world task. Thus, future research might replicate our results in a sample of police officers including the top-end of the ability bandwidth.

### Practical recommendations

From a practitioner perspective, police organizations might be predominately interested to assess individual differences in face processing abilities. Subsequently, police officers with superior face processing abilities might be deployed in tasks involving identity matching. Finally, performance in real-world police tasks, such as CCTV tasks, might be maximized. Importantly, to make sure that the high expectations in this emerging field are met, profound personnel selection processes are a necessary prerequisite (Robertson, 2018; Robertson et al., 2016, 2019a; Young & Burton, 2017). Following Ramon et al. (2019a), several issues might be ecologically important. At first, government practitioners are well-advised to collaborate closely with experts from the field of face processing, in order to close the gap between research and practice (Ramon, 2021; cf. Anderson et al., 2001). Recent personnel selection approaches such as the Berlin Model of SR identification seem to be promising in this regard (Ramon, 2021; Ramon & Rjosk, 2021; Rjosk, 2021). In this context, it is important to note that the focus of practitioners should not only rely on “super-recognizers.” Rather, the entire continuum of face processing abilities needs to be considered. Without empirical evidence on the relationship between laboratory test scoring and performance in real-world task, personnel selection practitioners can discuss pros and cons of selection decisions ranging from “selecting-out” inferior performance to “selecting-in” superior performance of face processing.

In this context, ecological relevant testing material needs to be derived from job analysis and well-defined job profiles of police officers (Sackett et al., 2012). Indeed, no studies to date seem to specify the ability levels of police officers required by police organizations (Moreton et al., 2019; Ramon et al., 2019a). From our point of view, police officers’ job profiles vary across different branches of police work (e.g., general protection police and special police forces). The tasks and the requirements concerning face processing abilities may significantly differ, if police officers wear uniform in highly standardized settings (e.g., person identification at border control), if they operate undercover in highly variable environments (e.g., observation in the field), or if they analyze CCTV footage. Thus, we would recommend to update job profiles of tasks that involve face processing (Robertson et al., 2019a). Indeed, job profiles might help to define both underlying relatively stable abilities and skills or characteristics that might be more responsive to job training and job experience (Ramon et al., 2019a). Based on job profiles and task analysis (Sackett et al., 2012), we would recommend to design ecological meaningful work samples that might be incorporated to test batteries in order to assess individual differences in face processing in the police context more validly (Robertson et al., 2019a).

Although the development of work samples to select personnel for specific jobs may cost a certain amount of effort, a positive cost to benefit ratio may still result. According to the Taylor–Russell model (1939) in personnel psychology, a high probability to select a true-positive employee is a function of three factors: the base rate (i.e., the proportion of applicants who meet the selection criteria), the selection rate (i.e., the number of applicants to be selected) and the validity of the assessment procedure. If an organization aims to select for a face recognition unit with a small number of specialized police officers with superior face processing abilities (i.e., low base rate and low selection rate), a high validity is needed to maximize the likelihood of true-positive selection decisions (cf. Taylor & Russell, 1939).

The present study revealed a relative low accuracy observed on the CCTV task in general. Indeed, the novice group missed out 57% of targets, whereas the experienced police officers missed out 47%. Moreover, experienced police officers were only slightly more likely to select a target (7.4 hits for novices versus 9.2 hits for experienced) than they are to select an “innocent“ bystanders (3.9 false alarms for novices versus 5.0 false alarms for police). This result may suggest a relatively low accuracy in CCTV tasks. However, several compensatory factors may increase accuracy in the field (e.g., higher investment of time per video, second review by another police officer or specific contextual information about the criminal case). In sum, practitioners should be aware of reduced certainty of target identification in CCTV tasks, particularly when investigations predominately rely on face processing abilities, and processing time of the material is limited. Presumably, low performance on average depends on different scene settings recorded. For efficient personnel selection, the underlying mechanisms of face processing deploy in CCTV need to be understood (Young & Burton, 2017).

However, we found a positive correlation of the CFPT+ test scores and performance in the real-world video task, indicating that better face recognizers as assessed by laboratory-based tasks, tended to perform better on this real-world task. This finding suggests that laboratory-based tests are useful to predict real-world performance in CCTV tasks and might be integrated in personnel selection processes. Finally, rather than focusing on mean performance, we are interested in superior performance. The best subjects’ performance score (sample 1: 24% missed targets; sample 2: 18% missed targets) in the two samples appeared to be far better than overall mean performance (sample 1: 57% missed targets; sample 2: 47% missed targets). Thus, effective personnel selection procedures helping to find the best performers might promote success of a specialized face recognition unit for crime prevention and law enforcement.

## Supplementary Information

**Additional file 1.** **Supplementray Table 4.** Hits, hit rates %, false alarms and item statistics of the targets of the CCTV task.

## Data Availability

The datasets generated for this study contain data of police officers belonging to a security-relevant government agency. Thus, the data cannot be made available online. The experimental materials of the CCTV task are primarily developed for the purpose of the research at hand. Subsequently, the actors involved only provided their consent with respect to the present investigation. We intend to develop open-access CCTV materials for other research groups. For details, please contact the corresponding author.

## Footnotes

1 Regarding the CFPT (Duchaine et al., 2007), we assumed that performance on the CFPT negatively predicted performance in the real-world CCTV task of person identification. Regarding the first sample, the CFPT score of upright faces negatively predicted hits of targets (*r* = − .20, *p* < .05, one-tailed; 95% CI [− .36, − .03]), but not false alarms (*r* = .14, *p* > .05, one-tailed, 95% CI [− .03, .31]). The CFPT explains 4.0% of variance in hits and 2.0% of variance in false alarms. Regarding the second sample, the CFPT score of upright faces negatively predicted hits of targets (*r* = − .35, *p* < .05, one-tailed, 95% CI [− .63, − .07]), but not false alarms (*r* = .16, *p* > .05, one-tailed, 95% CI [− .14, .46]). Neither the inverted face score, nor the difference score did not add further variance explanation (cf. Tardif et al., 2018). In sum, individuals with lower CFPT scores showed superior performance in the CCTV task. The CFPT explains 12.5% of variance in hits and 2.6% of variance in false alarms.
