# Supplementary material for: Face processing in police service: the relationship between laboratory-based assessment of face processing abilities and performance in a real-world identity matching task
Source: Cogn Res Princ Implic. 2021 Aug 5;6:54. doi: 10.1186/s41235-021-00317-x (PMC8342700; doi:10.1186/s41235-021-00317-x)
Supplement: Supplementary file 1 — Additional file 1. Supplementray Table 4. Hits, hit rates %, false alarms and item statistics of the targets of the CCTV task. [file 41235_2021_317_MOESM1_ESM.docx]

**Supplemental Material**

Table 4. *Hits, Hit Rates %, False Alarms and Item Statistics of the Targets of the CCTV Task.*

| Target | *Hits* | | | | *False Alarms* | | |
| --- | --- | --- | --- | --- | --- | --- | --- |
|  | *appeared*  *in videos* | Hits | Hit rate % | *Correlation Hits-CFMT+* | *did not appear  in Videos* | *False*  *alarms* | *Correlation F.A.-CFMT+* |
|  |  | *M* (*SD*) | *M* (*SD*) | *r* |  | ***M*** (*SD*) | *r* |
|  |  |  |  |  |  |  |  |
| A | K, M | .86 (.71) | .43 (.35) | .20* | C, D, E, F, G, H, I, J, L | .41 (.67) | -.12 |
|  |  | 1.00 (.66) | .50 (.33) | .23 |  | .49 (.72) | -.29* |
|  |  |  |  |  |  |  |  |
| B | D, J | .68 (.73) | .34 (.37) | .16 | C, E, F, G, H, I, K, L M | .79 (.81) | .07 |
|  |  | .89 (.76) | .45 (.38) | .24 |  | .72 (.80) | -.11 |
|  |  |  |  |  |  |  |  |
| C | C, M | .82 (.53) | .41 (.26) | .15 | D, E, F, G, H, I, J, K, L | .55 (.76) | -.06 |
|  |  | 1.04 (.55) | .52 (.28) | .09 |  | .64 (.76) | -.21 |
|  |  |  |  |  |  |  |  |
| D | F, L | 1.04 (.59) | .52 (.30) | .06 | C, D, E, G, H, I, J, K, M | .54 (.79) | -.06 |
|  |  | 1.30 (.66) | .65 (.33) | .19 |  | .79 (.86) | -.33* |
|  |  |  |  | . |  |  |  |
| E | H, K | .85 (.71) | .42 (.35) | .11 | C, D, E, F, G, I, J, L, M | .12 (.35) | .02 |
|  |  | 1.26 (.71) | .63 (.35) | .11 |  | .23 (.43) | -.05 |
|  |  |  |  |  |  |  |  |
| F | E, H | .39 (.57) | .19 (.29) | .08 | C, D, F, G, I, J, K, L, M | .43 (.72) | -.03 |
|  |  | .43 (.58) | .21 (.29) | .31* |  | .55 (.75) | -.15 |
|  |  |  |  |  |  |  |  |
| G | G | .42 (.50) | .42 (.50) | .05 | C, D, E, F, H, I, J, K, L, M | .31 (.55) | -.10 |
|  |  | .43 (.50) | .43 (.50) | .23 |  | .45 (.77) | .01 |
|  |  |  |  |  |  |  |  |
| H | G, I | 1.30 (.76) | .65 (.38) | .19* | C, D, E, F, H, J, K, L, M | .24 (.48) | -.03 |
|  |  | 1.68 (.52) | .84 (.26) | .17 |  | .40 (.74) | -.35* |
|  |  |  |  |  |  |  |  |
| I | J, L | 1.13 (.76) | .56 (.38) | .16 | C, D, E, F, G, H, I, K, M | .18 (.42) | .00 |
|  |  | 1.43 (.74) | .71 (.37) | .08 |  | .40 (.77) | -.21 |
|  |  |  |  |  |  |  |  |

*Notes*. The first row displays results of the first sample (*N* = 139), and the second row of the second sample (*N* = 47). Significant correlations are marked with ** *p* < .01, and * *p* < .05, respectively
